# Advances in the study of aerobic glycolytic effects in resistance to radiotherapy in malignant tumors

**DOI:** 10.7717/peerj.14930

**Published:** 2023-02-16

**Authors:** Yuyi Liu, Zhengfu Feng, Pingmei Zhang, Honghao Chen, Song Zhu, Xin Wang

**Affiliations:** Department of Radiotherapy, The Sixth Affiliated Hospital of Guangzhou Medical University, Qingyuan, China

**Keywords:** Metabolic reprogramming, Aerobic glycolysis, Cancer, Radioresistance

## Abstract

Aerobic glycolysis is a metabolic mode of tumor cells different from normal cells that plays an important role in tumor proliferation and distant metastasis. Radiotherapy has now become a routine and effective treatment for many malignancies, however, resistance to radiotherapy remains a major challenge in the treatment of malignant tumors. Recent studies have found that the abnormal activity of the aerobic glycolysis process in tumor cells is most likely involved in regulating chemoresistance and radiation therapy resistance in malignant tumors. However, research on the functions and mechanisms of aerobic glycolysis in the molecular mechanisms of resistance to radiotherapy in malignant tumors is still in its early stages. This review collects recent studies on the effects of aerobic glycolysis and radiation therapy resistance in malignant tumors, to further understand the progress in this area. This research may more effectively guide the clinical development of more powerful treatment plans for radiation therapy resistant subtypes of cancer patients, and take an important step to improve the disease control rate of radiation therapy resistant subtypes of cancer patients.

## Introduction

Professor Otto Warburg first demonstrated in 1927 that proliferating cancer cells can consume glucose to produce lactate even under aerobic conditions. This phenomenon is referred to as the Warburg effect or aerobic glycolysis (Warburg, Wind & Negelein, 1927). Research on metabolic mechanisms in malignant tumors has shown that the Warburg effect is an important manifestation of reprogramming of energy metabolism in malignant tumors, and this reprogramming of cellular metabolism is considered to be a hallmark of cancer. Accordingly, aerobic glycolysis is widely observed in malignant tumors and influences malignant biological behaviors such as proliferation, resistance to apoptosis, metastases, immune escape, and resistance to radiotherapy (Grotius et al., 2009; Quennet et al., 2006; Shimura et al., 2014).

Radiation therapy is an important treatment modality for many cancers, and 50% of cancer patients achieve remission or are cured with radiation therapy (Liu et al., 2021). The main mechanism of radiotherapy is the induction of tumor cell death by radiation-generated free radical-induced DNA damage. However, recent studies have found that intermediates of the aerobic glycolytic pathway in malignant tumor cells scavenge free radicals and reactive oxygen species (ROS) to induce resistance to radiotherapy (Yang et al., 2020; Sattler et al., 2010). In this article, we will describe the developments in the research on aerobic glycolysis and related targets in radiotherapy resistance of malignant tumors from two broad perspectives: differences between the glycolytic metabolism of malignant tumor cells and normal cells, and the role of aerobic glycolysis-related enzymes and products in radiotherapy resistance.

### Survey methodology

The literature cited in the text is based on the authors’ personal choice to reliably describe the methods described in the text. Searches were conducted in the Google Scholar, PubMed, and Wanfang databases to select appropriate references as examples. Different keywords that were appropriate for the search were used for each analyzed case. The final selection of references included studies on the relationship between the Warburg effect and resistance to radiotherapy in different malignancies.

### Differences in glucose metabolism between malignant and normal cells

Glucose is the main source of energy for mammalian cells, and glucose most commonly enters the cell through the facilitated diffusion of glucose transporter protein (Navale & Paranjape, 2016). In normal cells, once glucose enters the cell, it is phosphorylated to glucose-6-phosphate by hexokinase, which produces pyruvate molecules in the cytoplasm through an oxygen-independent glycolytic reaction (Lincet & Icard, 2015). Under normal aerobic conditions, most of the pyruvate is transported into the mitochondria *via* mitochondrial pyruvate carriers (MPCs) or *via* monocarboxylate transporters (MCTs) after conversion to acetyl CoA; it undergoes oxidative phosphorylation through the tricarboxylic acid cycle and electron transport chain to generate ATP, specifically, 32–34 ATP for each glucose molecule (McCommis & Finck, 2015; Payen et al., 2020). Under anaerobic conditions, the cell cannot rely on oxidative phosphorylation to balance its redox state. In such conditions, one glucose molecule is converted to two pyruvate molecules, which are preferentially converted to lactate in a reaction catalyzed by cell membrane lactate dehydrogenase (LDH) and result in a net gain of two ATP molecules (Rogatzki et al., 2015). In cells, pyruvate is almost completely dissociated into lactate and H+, and the accumulation of lactate leads to acidification of the cytoplasm and effectively inhibits further glycolysis (Trivedi & Danforth, 1966). In contrast, cancer cells maintain an abnormally high expression of lactate dehydrogenase (LDH), a key enzyme for aerobic glycolysis, and thereby, cancer cells are able to maintain a high rate of aerobic glycolysis even under conditions of oxygen sufficiency through redistribution of lactate in the tumor microenvironment. Aerobic glycolysis provides cancer cells with raw materials, such as nucleotides, amino acids, and lipids, required for their growth to meet the rapid proliferation demands of cancer cells (Warburg, Wind & Negelein, 1927; Shimura et al., 2014). Three promising targets that are involved in the regulation of cancer cell aerobic glycolytic metabolism, namely, LDH, lactate, and the lactate transporter protein MCT, have been shown to modulate radiosensitivity.

### Role of enzymes and products of aerobic glycolysis in resistance to radiotherapy in malignant tumors

#### Role of LDH

LDH is present in all mammalian cells, and the LDH molecule is a tetramer composed of four polypeptide chains of M and/or H. The M-type (muscle) chain is encoded by the LDHA gene, and the H-type (heart) is encoded by the LDHB gene. Five isozymes, LDH1 to LDH5, can be produced by five different combinations of M and H chains. LDH-1 (H4) consists of four H subunits, while LDH-5 (M4) consists of four M subunits. LDH-2, LDH-3, and LDH-4 correspond to the MH3, M2H2, and M3H combinations respectively. The higher the number of M subunits in the LDH molecule, the stronger is its ability to catalyze the conversion of pyruvate to lactate. In contrast, H-dominant isozymes catalyze the reverse conversion of lactate to pyruvate (Millar & Schwert, 1963). The most common isoform is LDHA, which preferentially reduces pyruvate to lactate and is frequently overexpressed in many tumors (de la Cruz-Lopez et al., 2019). Further, LDH is considered as an independent prognostic factor for several malignancies, and elevated serum LDH in the body often indicates poor prognosis in cancer patients (Fu et al., 2016).

Radiotherapy is one of the treatment options for malignancies, and in some malignancies, such as nasopharyngeal carcinoma and glioblastoma, radiotherapy is considered as the treatment of choice (Lutterbach, Sauerbrei & Guttenberger, 2003; Zhou et al., 2016). However, there is a lack of available data regarding the role of serum LDH in the response to radiotherapy. In a study of 318 patients with glioblastoma treated with radiation therapy (Lutterbach, Sauerbrei & Guttenberger, 2003), the results suggested that high serum LDH levels were significantly associated with poor patient prognosis—a finding that may indicate increased resistance to radiation in glioblastoma patients with high serum LDH levels. Local control of tumor growth was not analyzed or reported, so aggressive local tumor growth after recurrence could also explain this correlation. However, related studies have found that silencing the LDHA gene can reduce ATP production, inhibit cell growth, decrease invasiveness, and induce oxidative stress and radiosensitivity in cancer cells (Hou et al., 2019; Meng et al., 2015; Yang et al., 2021). Therefore, the development of LDH inhibitors has become a hot topic of discussion among researchers in this field, and several preclinical studies have analyzed the effects of selective and non-selective inhibitors of LDHA on cancer cells. For example, AT-101, a natural compound derived from cottonseed, is an orally administered non-selective inhibitor of LDH that also inhibits the anti-apoptotic proteins Bcl-2, Bcl-xL, Bcl-W, and Mcl-1 while stimulating pro-apoptotic signaling (Liu et al., 2009; Meng et al., 2008). By inhibiting LDH, AT-101 induces the production of ROS, promotes the release of cytoplasmic Ca2+ ions, and causes a reduction in the mitochondrial inner membrane potential, and this ultimately induces apoptosis in cancer cells and improves radiotherapy sensitivity. In particular, ROS is an important factor for the induction of tumor cell death *via* DNA damage caused by the ionizing effect of radiation. A study of AT-101 monotherapy in 23 men with metastatic desmoplastic-resistant prostate cancer showed that a dose of 20 mg/day was well tolerated and resulted in more than 50% decrease in PSA in 9% of the patients (Liu et al., 2009). Further, FX11, a derivative of AT-101, selectively inhibits LDHA but not LDHB, and in lymphoma and pancreatic tumor xenograft models, it effectively inhibited tumorigenesis (Meng et al., 2008). When combined with a small molecule inhibitor of nicotinamide adenine dinucleotide (NAD+), FX11 was able to further induce tumor regression in a lymphoma xenograft model (Le et al., 2010). In 2017, Michael Koukourakis’ team evaluated the expression of the LDH5 isoenzyme in human glioblastoma tissue, and their results showed that the enzyme is encoded exclusively by the LDHA gene. They also found that 20% of cancer cells exhibited strong positive expression of LDH5 in the cytoplasm and nucleus through immunohistochemical analysis (Koukourakis et al., 2017). In addition, their study showed that the LDHA inhibitor oxalate oxamate, when combined with the glycolysis inhibitor 2-deoxy-d-glucose, effectively enhanced the antitumor effects of temozolomide, improved the radiosensitivity of glioblastoma, and triggered apoptosis and differentiation of cancer stem cells (Koukourakis et al., 2017). Recently, a novel LDH metamorphosis inhibitor, PSTMB, was found to reduce cell proliferation in *in vitro* models of lung, breast, melanoma, liver, and colon cancers. Moreover, this novel LDH inhibitor was able to reduce LDH activity under both aerobic and anaerobic conditions without altering LDH expression and increasing ROS formation and was, thus, able to improve radiotherapy sensitivity (Kim et al., 2019). Therefore, based on the findings of these studies, PSTMB may be of value for future studies on radiosensitizers.

#### Role of the aerobic glycolysis product lactate

Lactate, the product of the glycolytic process, is a gluconeogenic precursor and a regulator of the cellular redox state. Under steady-state conditions, lactate is either used intracellularly or secreted into the extracellular space for use by neighboring cells (Brooks, 1985; Romero et al., 2015). In the exercising muscle, lactate is produced by fast contracting muscle fibers *via* glycolysis, exported extracellularly *via* monocarboxylate transporter protein 4 (MCT4), and then transported to the liver to be utilized as a substrate for gluconeogenesis or taken up by neighboring slow oxidizing fibers *via* MCT1 as a precursor to glucose to replenish energy stores. A similar mechanism has been observed in tumor cells (Sonveaux et al., 2008; Koukourakis et al., 2006). Under the regulation of an oxygen gradient, hypoxic tumor cells away from the anaerobic glycolysis product lactate, which is released by the cells *via* MCT4. Lactate then diffuses along the concentration gradient into tumor cells with high oxygen content within the tumor vasculature, enters the cells *via* MCT1, and is oxidized to produce energy through oxidative phosphorylation. The Dewhirst laboratory reported in 2008 that when MCT1 is inhibited, oxidative tumor cells are forced to use glucose for energy production, ultimately accelerating glucose consumption through “glucose starvation induction,” which contributes to tumor cell necrosis. Glucose starvation induction is a cystine-regulated pentose phosphate pathway-dependent and disulfide-inhibited reprogramming of cancer cell metabolism, and it is highly dependent on specific nutrients such as glucose (Sonveaux et al., 2008). These findings provide the first evidence of metabolic cooperation between aerobic and hypoxic tumor cells that leads to metabolic heterogeneity within the tumor structure. These results also demonstrate that cancer cells can opportunistically optimize the available metabolic substrates in their microenvironment to increase tumor survival and growth.

Ionizing radiation and some chemotherapeutic drugs kill cancer cells by inducing oxidative stress in target cells and stimulating the overproduction of ROS, thus leading to DNA and RNA damage, lipid peroxidation, and genomic instability. Moreover, ROS is necessary for the repair of radiation-induced DNA damage. Sattler et al. (2010) reported that 30 conventional fractionated radiotherapy treatments were administered to 10 head and neck squamous cell carcinoma transplant tumor tissues over 6 weeks, and this was followed by fluorescence imaging of tumor tissue before and after irradiation for measurement of lactate, pyruvate, glucose, and ATP levels. Their results showed that there was a positive correlation between lactate concentrations and the radiotherapy resistance of tumor tissue. This correlation may be attributable to the antioxidant properties of lactate, which confers resistance against radiation-induced DNA damage by reducing the oxidative stress exerted therapeutically by radiation through the accumulation of ROS (Hirschhaeuser, Sattler & Mueller-Klieser, 2011). Thus, the accumulation of antioxidants (*e.g*., lactate) may induce or enhance resistance to radiation and may lead to chemoresistance (Martinez-Outschoorn, Lisanti & Sotgia, 2014). A prospective study conducted over 15 years applied induced metabolic bioluminescence imaging to detect lactate levels in head and neck squamous cell carcinoma tissue and found that lactate levels were a predictor of tumor recurrence after radiotherapy (Blatt et al., 2016). It has also been demonstrated in animal studies that malignant tumors with high levels of lactate prior to treatment exhibit a decrease in serum lactate levels after chemotherapy or radiotherapy (Romero et al., 2015). These findings indicate that monitoring the levels of the aerobic glycolytic metabolite lactate in human tumors may be useful for predicting the therapeutic response of tumors to radiotherapy.

#### Role of the lactate transporter protein MCT

Tumor survival is dependent on the shuttling of lactate between cells, and the transporters mediating this process are MCT1 and MCT4. Therefore, these two MCTs are potential targets for enhancing the efficacy of radiotherapy. MCT is a class of plasma membrane transporters that carry lactate and pyruvate across biological membranes during glycolysis. Tumors with a high degree of malignancy rely primarily on glycolytic processes for anabolic metabolism and, therefore, require MCT transporter proteins to transport lactate from cancer cells to the extracellular tumor microenvironment and protect tumor cells from acidosis induced by excessive lactate accumulation; these processes promote tumor progression by regulating various parameters in the tumor microenvironment, including cell invasion, angiogenesis, survival signaling, metastatic development, and evasion of immune surveillance (Halestrap & Wilson, 2012). Accordingly, MCT1 (SLC16A1) and MCT4 (SLC16A3) have been reported to play important roles in tumors and are aberrantly highly expressed in several cancers, including breast, colon, pancreatic, glioblastoma, prostate, and renal cell carcinomas, and are involved in the development of antitumor drug resistance (Pinheiro et al., 2008; Fisel, Schaeffeler & Schwab, 2018). For example, in order to prevent cell death caused by lactate acidification in cancer cells, lactate is excreted from oxygen-deprived cancer cells *via* MCT4 and subsequently enters oxygen-rich cancer cells *via* MCT1, where it is used by cancer cells as an alternative feedstock for energy production (Park et al., 2018). Aerobic tumor cells contain lower levels of HIF-1, which results in inefficient glycolysis. Thus, in order to meet their energy requirements, aerobic tumor cells use lactate produced by hypoxic tumor cells within the same tumor. That is, as glucose consumption by aerobic tumor cells is reduced, more glucose becomes available to hypoxic tumor cells. This symbiotic relationship between tumor cells enhances their ability to survive and proliferate (Payen et al., 2020; Doherty & Cleveland, 2013). *In vitro* experiments on the treatment of oral squamous cell carcinoma cell lines with MCT1/MCT4 inhibitors combined with radiotherapy showed that the proliferation, migration, and cladogenesis of the cancer cells were significantly reduced; thus, these findings demonstrate that MCT1/MCT4 inhibitors and radiotherapy have a synergistic anti-tumor effect (Brandstetter et al., 2021). Further, in an animal experiment in which combined therapy was compared with radiotherapy alone, tumor-bearing mice were administered AZD3965 orally (100 mg/kg, two times a day) for 7 consecutive days and were treated with conventionally fractionated radiotherapy (total, 6 Gy) that was started on day 3, while the control group received only radiation therapy. The rate of tumor tissue shrinkage, the survival time of mice, and the lactate content in transplanted tumor tissues were subsequently measured, and the results showed that mice treated with the MCT1 inhibitor in combination with radiotherapy had significantly smaller tumors, longer survival time, and higher intratumoral lactate levels than mice in the radiotherapy alone group. Thus, using the MCT1 inhibitor AZD3965 in combination with radiotherapy has better antitumor effects than administering radiotherapy alone, and AZD3965 may, therefore, have clinical potential as a radiosensitizer (Bola et al., 2014). These findings suggest that monitoring MCT1/MCT4 levels in human tumors may be useful for predicting the efficacy of tumor radiotherapy. In addition, the development of inhibitors targeting MCT1 may help enhance the antitumor effects of radiotherapy (Table 1).

**Table 1:** Summary of clinical studies of selected aerobic glycolytic targets and their inhibitors in cancer radiation therapy.

Target points	Inhibitors	Types of cancer	References
LDH	AT-101	Prostate cancer	Liu et al. (2009)
LDH	Oxamate	Glioblastoma	Koukourakis et al. (2017)
MCT1	AZD3965	Small cell lung cancer	Bola et al. (2014)
MCT1/MCT4	AR-C122982	Squamous cell carcinoma of the oral cavity	Brandstetter et al. (2021)
MCT1/MCT4	AR-C155858	Squamous cell carcinoma of the oral cavity	Brandstetter et al. (2021)
MCT1/MCT4	simvastatin	Squamous cell carcinoma of the oral cavity	Brandstetter et al. (2021)
MCT1/MCT4	2-cyano-3-(4-hydroxyphenyl)-2-propenoic acid	Squamous cell carcinoma of the oral cavity	Brandstetter et al. (2021)

## Conclusions

Taken together, the findings of the reviewed studies suggest that aerobic glycolysis has an important role in resistance to radiation therapy in malignant tumors. Specifically, the metabolic conversion of tumor cells by aerobic glycolysis to produce intermediates that increase cell growth and division is a very early oncogenic event. Further, lactate, an important product of aerobic glycolysis, promotes tumor growth, in addition to being a source of energy. Elevated lactate levels and lactate-mediated downstream pathways have been found to cause changes in transcription (Dang et al., 2009; Ye, Guan & Xiong, 2018), tumor immune microenvironment (Yang et al., 2020), and lipid synthesis (Beloueche-Babari et al., 2020). Importantly, lactate can reduce ROS levels in tumors through these downstream changes, thereby reducing the tumor cell death induction ability of radiotherapy and promoting cancer cell resistance to radiotherapy (Hirschhaeuser, Sattler & Mueller-Klieser, 2011). In line with these findings, inhibitors of LDH and MCT, which regulate aerobic glycolytic metabolism, have been found to have potential as radiosensitizers in models of glioblastoma, pancreatic, lung, and cervical cancer (Yang et al., 2020; Koukourakis et al., 2017; Bola et al., 2014; Corbet et al., 2018). However, clinical studies of the combined application of these inhibitors with radiation are still limited. Further research is needed in the future to understand the mechanisms by which aerobic glycolytic modulators regulate tumorigenesis, to identify tumor subtypes that rely on the aerobic glycolytic pathway, and to further explore targeted inhibitors of this pathway in preclinical and clinical studies. Furthermore, the high degree of malignancy of tumors with high rates of aerobic glycolysis is indicated through markers of biochemical and pathophysiological mechanisms (Warburg, Wind & Negelein, 1927; Shimura et al., 2014). However, it remains unclear why seemingly identical tumors exhibit extreme differences in their tissue lactate content (Sattler et al., 2010; Liu et al., 2009; Koukourakis et al., 2017; Brandstetter et al., 2021). This is another challenge for future research in this field.

In conclusion, energy metabolic conversions caused by aerobic glycolysis in cancer cells not only represent promising molecular markers for monitoring sensitivity to radiotherapy in malignancies, but also provide new targets for the development of new cancer treatment strategies. Importantly, these findings open an exciting new chapter of research in the field of resistance to radiotherapy in malignancies.

## Additional Information and Declarations

### Data Availability

The following information was supplied regarding data availability: The raw data are available in the Table.
